# Microbial Metabolites of 3-*n*-butylphthalide as Monoamine Oxidase A Inhibitors

**DOI:** 10.3390/ijms241310605

**Published:** 2023-06-25

**Authors:** Joanna Gach, Joanna Grzelczyk, Tomasz Strzała, Filip Boratyński, Teresa Olejniczak

**Affiliations:** 1 Department of Food Chemistry and Biocatalysis, Wrocław University of Environmental and Life Sciences, Norwida 25, 50-375 Wrocław, Poland; filip.boratynski@upwr.edu.pl; 2 Institute of Food Technology and Analysis, Faculty of Biotechnology and Food Sciences, Lodz University of Technology, Stefanowskiego 2/22, 90-924 Łódź, Poland; joanna.grzelczyk@p.lodz.pl; 3 Department of Genetics, Wrocław University of Environmental and Life Sciences, Kożuchowska 7, 51-631 Wrocław, Poland; tomasz.strzala@upwr.edu.pl

**Keywords:** 3-*n*-butylphthalide, monoamine oxidase A inhibitor, biotransformation, serotonin, fungal strains

## Abstract

Novel compounds with antidepressant activity via monoamine oxidase inhibition are being sought. Among these, derivatives of 3-*n*-butylphthalide, a neuroprotective lactone from *Apiaceae* plants, may be prominent candidates. This study aimed to obtain the oxidation products of 3-*n*-butylphthalide and screen them regarding their activity against the monoamine oxidase A (MAO-A) isoform. Such activity of these compounds has not been previously tested. To obtain the metabolites, we used fungi as biocatalysts because of their high oxidative capacity. Overall, 37 strains were used, among which *Penicillium* and *Botrytis* spp. were the most efficient, leading to the obtaining of three main products: 3-*n*-butyl-10-hydroxyphthalide, 3-*n*-butylphthalide-11-oic acid, and 3-*n*-butyl-11-hydroxyphthalide, with a total yield of 0.38–0.82 g per g of the substrate, depending on the biocatalyst used. The precursor–3-*n*-butylphthalide and abovementioned metabolites inhibited the MAO-A enzyme; the most active was the carboxylic acid derivative of the lactone with inhibitory constant (K_i_) < 0.001 µmol/L. The in silico prediction of the drug-likeness of the metabolites matches the assumptions of Lipinski, Ghose, Veber, Egan, and Muegge. All the compounds are within the optimal range for the lipophilicity value, which is connected to adequate permeability and solubility.

## 1. Introduction

Depression is a disease with many faces that affects people of all ages. There are several hypotheses regarding its etiology [1]. The most described and valid theory in the literature is the monoamine hypothesis, which assumes that depression is caused by the dysfunction of serotonergic (5-HT) and noradrenergic pathways [2]. In addition to reduced levels of 5-HT, dopamine, and norepinephrine in various areas of the brain (e.g., hippocampus, amygdala), there may be a genetic mutation of the 5-HT transporter and the amino oxidase (MAO) enzyme, which is associated with gene polymorphism and passed from mother to child [2,3]. This disease can affect people with dementia and those on the autistic spectrum or with bipolar diseases [1,2,3,4,5]. Monoamine oxidase belongs to the family of flavoenzymes located in the mitochondrial membrane [6]. There are two isoforms, MAO-A and MAO-B, which differ in affinity to the substrate and susceptibility to inhibition [7]. Generally, the enzymes are responsible for the deamination of neurotransmitters [6,8]. MAO-A is the main enzyme that deaminates 5-HT in the brain, affecting behavioral disorders [9]. Almost all MAO-A inhibitors on the market act irreversibly and require dietary restrictions to avoid tyramine accumulation, leading to serotonin toxicity syndrome [7]. The only reversible commercially available drug that regulates MAO is moclobemide [7,10]. Therefore, there is a necessity for novel, non-toxic compounds that would regulate the level of serotonin in the human body.

The compound 3-*n*-butylphthalide is a biologically active lactone found in plants from the celery family [11,12]. Originally an ingredient in traditional Chinese medicine, it was accepted in China as a drug for cerebral ischemic stroke that improves the neurological function of the patients [13,14,15]. The compound restores microcirculation and protects the mitochondria. It shows vasodilatory, antiplatelet, and anti-inflammatory activity [15,16,17]. The antidepressant properties of 3-*n*-butylphthalide have also been studied [18]. The available literature outlines the positive effect of 3-*n*-butylphthalide in lipopolysaccharide (LPS)-induced depression [19] among chronic-stressed rats by the alteration of the serotonergic system, BDNF-ERK-mTOR signaling [18], and other mechanisms [20].

According to the research conducted by Diao et al. [21], the lactone in the human body is rapidly metabolized to 23 compounds, and mainly hydroxyderivatives and glucosidation products are observed. Although 3-*n*-butylphthalide has been widely tested regarding its biological activity, information on the monoamine oxidase A (MAO-A)-inhibiting properties of its metabolites is lacking. Moreover, other biological activities of these phthalide metabolites remain undiscovered, as obtaining them in sufficient amounts for research often poses a challenge. Biotransformation using whole fungal cells may provide a solution to this issue. Filamentous fungi feature many CYP isoenzymes and are, therefore, particularly known for their oxidative capacity. As a result, these biocatalysts lead to the formation of aliphatic and aromatic hydroxy derivatives [22]. Fungal-mediated biotransformations have been exploited by Diao et al. [21] for 3-*n*-butylphthalide and by us for its derivative with an unsaturated bond in the side chain [23].

Thus, this study aimed to obtain human metabolites of 3-*n*-butylphthalide through biotransformation by whole cells of filamentous fungi. After obtaining the oxidation products, we tested them in terms of their MAO-A enzyme-inhibiting properties. In addition, selected pharmacokinetic parameters were investigated using bioinformatics tools to assess the suitability of the compounds as potential antidepressant drugs.

## 2. Results

### 2.1. Chemical Synthesis of 3-n-butylphthalide (***1***) and Biotransformations

**Figure 1:**
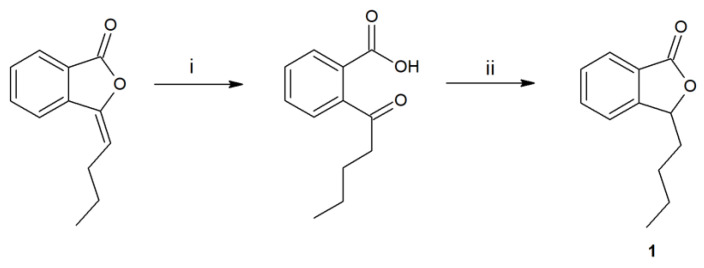
Synthesis of 3-*n*-butylphthalide (**1**) by the hydrolysis of 3-*n*-butylidenephthalide to ketoacid (i-KOH in MeOH), its reduction by borohydride, and acidification (ii-THF, NaBH_4_, HCl).

The first step of the research was the synthesis and purification of substrate **1**, obtained in a two-step process (Figure 1). 

After obtaining the 3-*n*-butylphthalide (**1**), the screening of whole fungal cells was carried out. We searched for biocatalysts capable of efficiently converting 3-*n*-butylphthalide (**1**) to oxidation products. During the biotransformation processes, we collected samples 4, 8, and 12 days after adding the substrate. After extraction with ethyl acetate, we performed high-performance liquid chromatography-diode array detector (HPLC-DAD) analyses.

**Figure 2:**
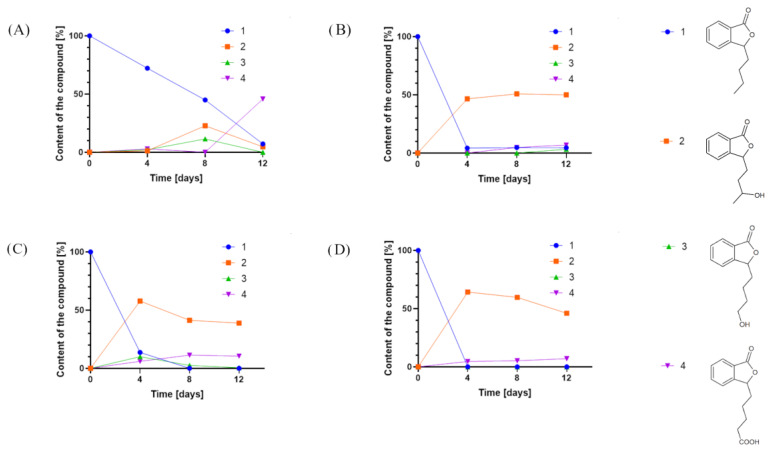
Product content of 3-*n*-butylphthalide (**1**) biotransformations catalyzed by (**A**) *Penicillium dierckxii* AM32, (**B**) *Penicillium* sp. AM91, (**C**) *Botrytis cinerea* AM235, and (**D**) *Botrytis* sp. KKP3292, determined by an HPLC-DAD at a wavelength of 274 nm.

Overall, we tested 37 fungal strains, and 12 (*Fusarium culmorum* AM7, *Sclerophoma pythiopila* AM55, *Spicaria fusispora* AM136, *Beauveria bassiana* AM278, *Pleurotus ostreatus* AM482, *Aspergillus niger* strains KKP45, KKP423, KKP424, *Aspergillus flavus* KKP686, KKP689, *Phanerochaete chrysosporium* KKP784, and *Trichoderma lignorum* KKP786) did not transform the substrate. In the other biotransformation processes, we observed products formed from 3-*n*-butylphthalide (**1**) (Figure 2).

In most cases, the application of whole cells of fungi resulted in product **2** as the predominant product. Interestingly, during the biotransformations catalyzed by *Penicillium dierckxii* AM32, *Botrytis* sp. KKP3292, and *Botrytis cinerea* AM235, we observed a decrease in content during the biotransformation. Product **3** was observed using *Penicillium dierckxii* AM32, *Penicillium* sp. AM91, and *Botrytis cinerea* AM235. An almost total conversion of the starting substrate (**1**) was noted using both *Botrytis* strains as the catalysts.

**Figure 3:**
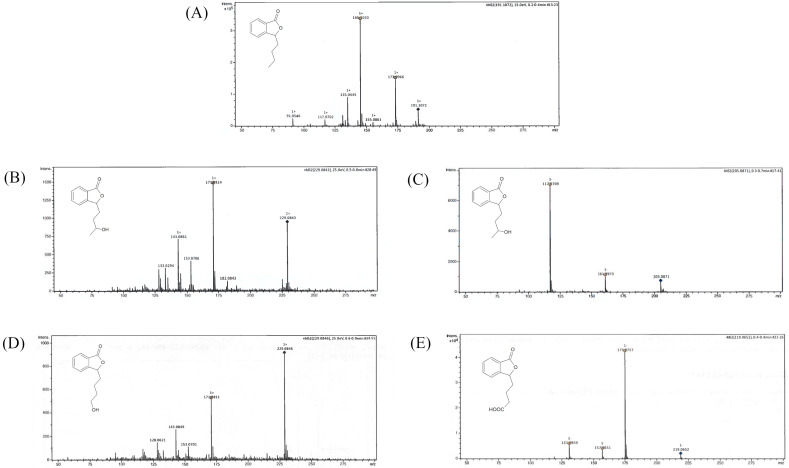
LC/ESI–MS/MS chromatograms of (**A**) 3-*n*-butylphthalide (**1**)—positive ion mode, (**B**) 3-*n*-butyl-10-hydroxy-phthalide (**2**)—positive ion mode, (**C**) 3-*n*-butyl-10-hydroxy-phthalide (**2**)—negative ion mode, (**D**) 3-*n*-butyl-11-hydroxy-phthalide (**3**)—positive ion mode, and (**E**) 3-*n*-butylphthalide-11-oic acid (**4**)—negative ion mode.

We conducted the upscale processes to determine the structure of products **2**–**4**. The biotransformations with biocatalysts AM91 and AM235 were conducted in the bioreactor at a concentration of substrate (**1**) equaling 0.4 g/L, while that with AM32 was conducted in a 2 L flask. After extraction with ethyl acetate and evaporation, the mixtures were purified by column chromatography on silica gel using gradient elution, gradually increasing the concentration of polar solvents. The structures of compounds **2**–**4** were determined based on nuclear magnetic resonance (NMR) spectroscopy and confirmed by high-resolution electrospray ionization mass spectrometry (HRMS). Figure 3 shows the LC/ESI–MS/MS chromatograms, while Table 1 presents information about the quasi-molecular ions, the proposed elemental formula, and fragment ions of compounds **1**–**4**, both from the experiments and from the literature. The NMR spectra for compounds **1**–**4** are presented in the Supplementary Materials (Figures S1–S8).

**Figure 4:**
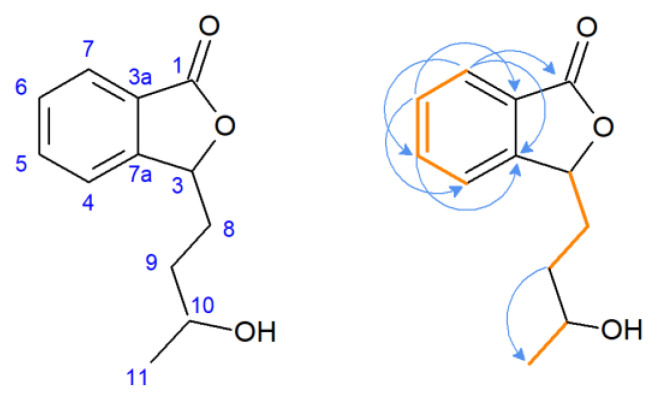
Pivotal couplings for compound **2**—correlation spectroscopy (orange) and nuclear multiple bond coherence (blue arrows).

The ^1^H NMR spectra of compound **2** revealed duplicated peaks in the area of 1.40–5.60 ppm corresponding to protons in the side chain of the lactone, which suggested the presence of two diastereoisomers. The characteristic signal of substrate **1** for proton H-3 (δ = 5.47 ppm) was present, excluding the possibility of hydroxylation at the C-3 position, the predominant direction of biotransformation of 3-*n*-butylidenophthalide found in our previous study [23]. Correlation spectroscopy (COSY) revealed the coupling of H-5 with H-6 and H-7, and H-6 with H-7 in the aromatic region (Figure 4). Other key couplings between the protons in the side chain were H-8 with H-3 and H-9 and H-10 with H-11. The most characteristic signal of the carbon spectrum was a downfield shift of C-10 carbon in relation to the substrate (22.6 ppm) corresponding signal. Instead, signals appeared at δ = 81.18 and δ = 81.68 ppm (two signals of the isomers). 

Compound **2** ionized in negative and positive modes, resulting in the formation of [M + H]^−^ 205.0863 and the adduct [M + Na]^+^ 229.0844, respectively. Fragment ion 171.0804 was formed after the loss of two water molecules. Product **2** was confirmed as 3-*n*-butyl-10-hydroxyphthalide (**2**).

The characteristic of the ^1^H NMR spectrum of compound **3** is the presence of a triplet at δ = 3.64 ppm of the integration, indicating the presence of two protons. The ^13^C NMR spectrum of **3** is similar to that of the 10-hydroxylated derivative (**2**), with a slight shift upfield of the signals δ = 21.36, 32.39, and 62.58 ppm. Compound **3** ionized in positive mode, and the 229.0844 [M + Na]^+^ adduct was formed with a similar fragment ion 171.0804 as that for product **2**. Product **3** was confirmed as 3-*n*-butyl-11-hydroxyphthalide.

In the ^1^H NMR spectrum of compound **4**, we observed the shift of the proton signals concerning the side chain of lactone towards the lower field compared to that of the substrate (**1**) and the product with a hydroxyl group at the C-10 position. The total integration of multiplets in the areas 1.74–1.88, 2.10–2.20, and 2.37–2.50 ppm indicated the presence of six protons. In the carbon spectrum, there were only three signals in the area characteristic for the side chain (14–30 ppm), with a lack of signal characteristic for C-11 in the substrate **1** spectrum (at δ = 14.0 ppm). However, we observed a new strongly de-shielded signal at δ = 179.8 ppm. The product ionized in the negative mode, leading to the formation of [M + H]^−^ 219.0650. The main fragment ions included 175.0765, formed after the loss of −CO_2_, 157.0659 (after the loss of −CO_2_ and −H_2_O), and 131.0866. Product **4** was identified as 3-*n*-butylphthalide-11-oic acid.

During the purification of the crude mixture after biotransformation, we isolated pure fractions of products **2**–**4** and mixtures of these compounds enriched with metabolites. As we had doubts concerning the assessment of the process efficiency, we conducted additional experiments to assess the percentage of substrate and products in the mixtures. For this purpose, biotransformations were performed with 3-*n*-butylphthalide (**1**) at a concentration of 260 mg per 1000 mL of culture. The cultures were extracted with ethyl acetate after 12 days for *Penicillium* sp. AM91, *B. cinerea* AM235, and *Botrytis* sp. KKP3292, and after 6 days for *Penicillium dierckxii* AM32. We prolonged the extraction time using new portions of solvent until no substrate or product remained in the extract. After the extraction, the amounts of the remaining substrate and the individual products **2**–**4** were assessed using gas chromatography coupled with a flame-ionization detector (GC-FID) using calibration curves from previously purified products. The obtained yields in relation to the biocatalyst are presented in Table 2.

Similar to the screening scale biotransformations, compound **2** was the most dominant metabolite for strains *B. cinerea* AM235 and *Botrytis* sp. KKP3292. *Botrytis* sp. KKP3292 led to an almost total conversion of substrate **1**. However, *Penicillium* sp. AM91, applied as a biocatalyst, led to the obtainment of, predominantly, compound **3**. AM32 led to the obtainment of mainly products **3** and **4**, and their relative ratio changed with the prolonged biotransformation time in favor of **4** (from 2:1 to 18:1 in biotransformation after 6 and 12 days, respectively).

### 2.2. Inhibition of MAO-A Activity

Protein-ligand interaction analysis was performed using isothermal titration calorimetry (ITC). During the analysis, the heat of uptake or release, due to the interaction of MAO-A with the tested compounds, was recorded. In this way, raw data were obtained in the form of exothermic heat versus time graphs. Additionally, the ligand was injected into 1% DMSO with methanol to subtract the energetic effects of the dilution of the ligand solution. The obtained thermodynamic parameters are presented in Table 3. The highest negative change in enthalpy was characterized by the interaction of the enzyme with compounds **3** (−19.19 kJ/mol) and **4** (−13.37 kJ/mol), and the lowest value of ΔH was recorded during interactions with compound **1** (−4.51 kJ/mol). The paired compounds caused a decrease in enthalpy while maintaining the exothermic effect of the reaction; the enthalpy ranged from −3.80 kJ/mol (**1** with **2**) to −8.28 kJ/mol (**3** with **4**). Affinity (ΔG) values were more evenly distributed between compound **1**, compounds **1** with **4**, **2** with **4**, and **2** with **3**. The lowest affinities were characterized by 1 with **3** and **1** with **2**, amounting to −20.69 and 22.69 kJ/mol, respectively, and the highest one was compounds **4**, amounting to −28.42 kJ/mol. The ability of the ligands to inhibit BChE activity was described on the basis of the IC_50_ parameter. The lowest IC_50_ value was shown by compound **3** (1.29 μmol/L), and the highest by compound **1** with **2** (45.00 μmol/L).

### 2.3. Absorption, Distribution, Metabolism, Excretion, and Toxicity (ADMET) Profiling

Substrate **1** and products **2**–**4** were tested for their pharmacokinetic properties, using the bioinformatic SWISS ADME tool reported by Daina et al. [25], including lipophilicity, solubility, and inhibition of the selected isoenzymes and drug-likeness. Toxicity to rat cells after oral administration was determined using the Way2Drug platform described by Druzhilovskiy et al. [26] (Table 4).

Lipophilicity is the distribution of the drug between the organic and water phases [27]. Biotransformation led to the obtaining of more polar and less lipophilic metabolites **2**–**4** compared to their precursor **1**. Among these, the most soluble metabolite in water was 3-*n*-butylphthalide-11-oic acid (**4**).

There are several selection criteria for predicting the potential drug properties, including Lipinski, Ghose, Veber, Egan and Muegge [28,29,30,31,32], describing the optimal number of carbon atoms, heteroatoms, rings, hydrogen bond donors and acceptors, rotatable bonds, the range for lipophilicity, molar refractivity, total polar surface, and molecular weight [33]. According to the predictions, compounds **2**–**4** match the objectives described by these researchers [28,29,30,31,32]; however, their precursor, 3-*n*-butylphthalide (**1**) does not meet the criterion of Muegge.

Interestingly, metabolism led to an increase in toxicity as the half-lethal dose of compounds **2**–**4** declined. The half-lethal dose for precursor (**1**) was 4872 mg/kg. The least toxic of the metabolites was 3-*n*-butyl-10-hydroxyphthalide (**2**), while 3-*n*-butylphthalide-11-oic acid (**4**) was twice as toxic to rat cells than 3-*n*-butylphthalide (**1**). 

The inhibition of CYP isoenzymes leads to drug interactions, causing side effects [34,35]. In silico tests were performed for the five isoforms, accounting for almost 90% of the metabolic reactions [35]. They revealed no inhibition of lactones **1**–**4** against CYP2C19, CYP2C9, CYP2D6, and CYP3A4. CYP1A2 was inhibited only by the precursor, 3-*n*-butylphthalide (**1**). This isoform is crucial for the inhibition of many drugs, including Alzheimer’s and Parkinson’s disease medications and analgesics [36]. 

### 2.4. Molecular Species Identification

The PROTAX-fungi tool results of the ITS fragment for the analyzed samples are shown in Figure S9. For the AM32 strain, the results indicate that the analyzed sample belongs to *P. dierckxii* (Figure S9, part A). The ITS results for the AM91 strain did not identify the systematic affiliation, as the results showed an 18% probability for unknown species and a 14% probability for *P. spinulosum* and *P. thomii* (Figure S10, part B). The AM235 strain was identified as *Botrytis cinerea* (Figure S9, part B). The ITS sequences for AM32 and AM235 were deposited in the National Center for Biotechnology Information (NCBI) database under accession number OQ875855 for AM32 and OQ875856 for AM235.

The genome sequence obtained for strain AM91 (after base-calling) had 1.64e + 09 bases with N50 = 1.23 kbp and median PHRED = 10.931. The Flye assembly results were 35,547,927 bp of total genome length with 91 fragments (N50 = 3,603,452 bp and longest fragment 7,742,766 bp) and a mean coverage of 28. Medaka analyses did not improve genome assembly in terms of length. Finally, the assembled genome was deposited in the NCBI database under accession number SAMN34128561.

## 3. Discussion

**Figure 5:**
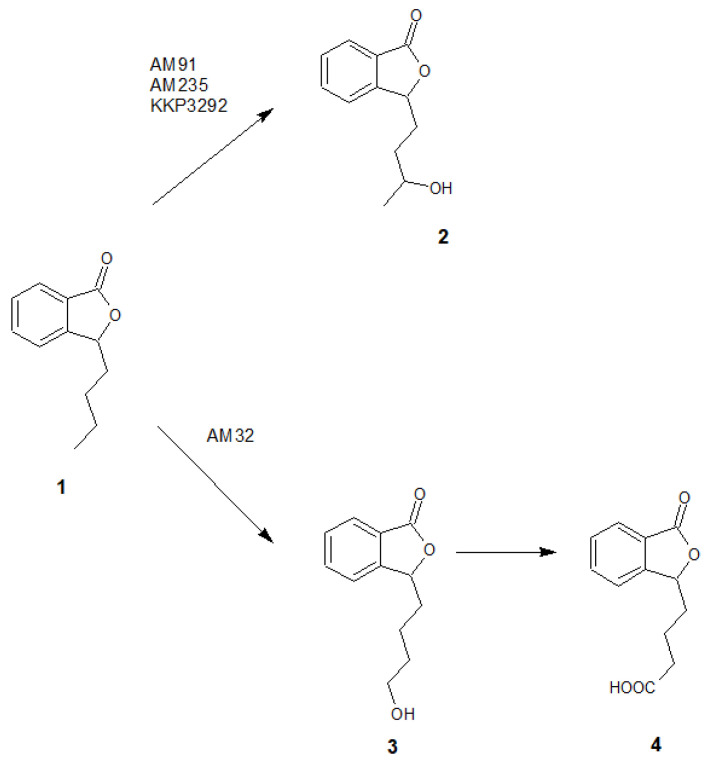
The structures of the main products formed during fungal-mediated biotransformations of 3-*n*-butylphthalide (**1**)—3-*n*-butyl-10-hydroxyphthalide (**2**), 3-*n*-butyl-11-hydroxyphthalide (**3**) and 3-*n*-butylphthalide-11-oic acid (**4**).

Based on the results of chromatographic analysis, we selected four fungal strains that allowed high substrate (**1**) conversion, including *Penicillium dierckxii* AM32, *Penicillium* sp. AM91, *Botrytis cinerea* AM235, and *Botrytis* sp. KKP3292, and we observed three main products. Notably, several *Penicillium* spp. have been used previously to convert one of the phthalides, mycophenolic acid, to the corresponding hydroxyderivative [11]. The hydroxylation of piperitone occurred in *Botrytis* AM235 [37]. The structures of the obtained main products depending on the biocatalyst are presented in Figure 5.

Diao et al. reported that the main direction of 3-*n*-butylphthalide (**1**) metabolism using human liver microsomes was the hydroxylation of the side chain. In vivo studies showed that 3-*n*-butyl-10-hydroxyphthalide (**2**) was the dominant circulating human metabolite, followed by the carboxylic acid derivative **4**, the hydroxylation product in the C-3 position, and the keto derivative. The biocatalysis of 3-*n*-butylphthalide (**1**) by whole cells of the *Cunninghamella blakesleana* ATCC9244 strain led to the formation of the products mentioned above; however, their ratio was not stated [21]. Moreover, the research conducted by Lin et al. confirmed that 3-*n*-butyl-10-hydroxyphthalide (**2**) was the main metabolite in the rat plasma and liver microsomes [38].

In our research, whole-cell biotransformations, similar to the in vivo tests, resulted in the obtaining of, primarily, the derivative in the C-10 position. Interestingly, there are no articles on the formation of two diastereoisomers of compound **2** during biotransformation; however, their presence was confirmed in the extract of *Ligusticum chuanxiong* [12]. Based on the decreasing content of C-11 hydroxyderivative (**3**) in favor of the 3-*n*-butylphthalide-11-oic acid (**4**) and the research conducted by Diao et al. [21], it can be confirmed that 3-*n*-butyl-11-hydroxyphthalide (**3**) is the mediatory product and is further oxidized to compound **4**.

Compounds **1**–**4** were tested for their inhibition of MAO-A activity by the ITC method. In the first stage, we assumed interactions between compounds **1**–**4** and the MAO-A enzyme to be potential ligands protecting against 5-HT degradation. Considering that a mixture of compounds may act differently than a single substance, with possible synergistic, additive, and antagonistic effects, we also tested compounds **1**–**4** in combinations [39,40]. The interactions between **1**–**4** and the MAO-A enzyme showed an exothermic effect, evidenced by negative enthalpy changes. An analysis of the stoichiometry of the resulting complexes showed a single 1:1 reaction-binding model [41]. During the analysis, there was a decrease in heat emitted after subsequent injections, which reflects the observed negatively charged compounds or mixtures in compounds, while interactions **1** and **2** with MAO-A were so weak that ITC could detect them. The change in the free enthalpy (affinity ΔG) indicated negative values, which characterizes the spontaneous nature of interactions. The values of individual compounds ranged from −28.42 to −23.49 kJ/mol for **4** and **3** associated with MAO-A complexation and blocking. The pairing of the compounds resulted in the stabilization of affinity at approximately −27 kJ/mol for pairs **1** with **4**, **2** with **3**, and **2** with **4**. However, for the remaining pairs, the affinity decreased, which may be related to the change in the energetic effect resulting from the rearrangement of the protein structure. 

The compounds with the most significant exothermic effects were **3** and **4**, suggesting the most significant conformational changes of MAO-A. In addition to complexation with active sites, exothermic changes were observed; therefore, injections of these compounds may limit the ability to deviate 5-HT. According to Prah, the calculated 5-HT degradation barrier is 62 kJ/mol, while the reaction catalyzed by the enzyme is reduced to −35.42 kJ/mol at pH 7.5. However, the same reaction in an aquatic environment shows a lower free energy of −22.06 kJ/mol, which means that the compounds are less stable in the aquatic environment than in pH 7.5 [42]. Our research confirms that the most stable bonds with the enzyme are characterized by compound 4 and the pairs of compounds **2** with **4**, **1** with **4**, and **2** with **3**. The values of the binding constant of the resulting complexes ranged from 3.12 × 10^3^ to 50 × 10^3^ L/mol. The significant differences in the height of K_A_ may be because some of the compounds have deprotonated, and the resulting electrostatic interaction affects the binding constant of these compounds with the enzyme. The highest MAO-A bond constant was characterized by the pair of compounds **2** and **4**, amounting to 50 × 10^3^ L/mol. In all cases, the values of free enthalpy and enthalpy of interactions were negative, and the entropy values were positive, indicating the formation of a ligand–protein complex through non-covalent hydrophobic interactions [43]. 

The tested compounds demonstrated the ability to reduce the rate of 5-HT deamination by the MAO-A enzyme. The IC_50_ was lowest for compound **3**, and the pair of compounds **3** and **4**, at 1.29 μmol/μmol of enzyme and 2.02 μmol/μmol of enzyme, respectively. Compounds **4** and **4** combined with **2** were at a similar level of about 5 μmol/μmol of the enzyme. Substrate **1**, tested with lactones **2** and **3**, turned out to be the least beneficial compounds, with a high concentration inhibiting the activity of the enzyme by 50%, and had the least favorable affinity. This effect can be explained by the binding of several compounds in the mixture to different MAO-A residues at the active site and in other regions. Molecular docking can be used in future research to confirm this assumption [44,45].

The K_i_ of the tested compounds indicates that they bound to MAO-A as competitive inhibitors and were characterized by a fairly similar and low K_i_ value for compounds **3** and **4** combined with **2**, amounting to <0.001 μmol/L. Various phthalide analogs have been tested regarding their MAO-inhibiting activity. According to Strydom et al., the K_i_ values for 6-phenyl-propoxyphthalide and CF_3_-substituted benzyloxyphthalide, the most active phthalide derivatives regarding MAO-A and MAO-B inhibition, were 0.062 µM and 0.0007 µM, respectively [46]. Qiang et al. tested the 3-*n*-butylphthalide–Edaravone complexes, that showed an inhibitory effect at IC_50_, in the ranges of 4.40–19.32 µM [47]. 

Absorption, distribution, metabolism, excretion, and toxicity are key in drug development [48]. The increase in lipophilicity is related to higher permeability through membranes and low solubility [49]. The values of this parameter for both butylphthalide and its metabolite were within the optimal range of logP, described as logP 1–3, ensuring their sufficient permeability and solubility [50]. The drug-likeness prediction for metabolites **2**–**4** revealed their obedience to the most common set of guidelines described by Lipinski, Ghose, Webber, Egan, and Muegge. However, 3-n-butylphthalide (**1**) showed incompatibility with the drug-likeness concept, according to Muegge, for the molecular weight, which is under 200. 

The predictions indicated the increased toxicity of the metabolites **2**–**4** compared to that of their precursor **1**. According to the research of Xue et al., the compounds phthalide **1** and their metabolites **2**–**4** were not toxic for primary rat hepatocytes and primary human hepatocytes tested at a concentration of 0–500 µM. However, the repetitive administration of **1** caused a reduction in cell viability [51]. Considering that toxicity is the second cause for clinical failures of drug development, it is crucial to conduct further preclinical studies on the metabolites **2**–**4** [52].

## 4. Materials and Methods

### 4.1. General

The content of the products was monitored using an HPLC—Dionex UltiMate 3000 instrument with a diode array detector (Thermo Fisher Scientific, Waltham, MA, USA) with a column Agilent Zorbax Bonus-RP 3.5 µm 150 × 3 mm. The mobile phase was composed of water acidified with 0.5% formic acid (A) and acetonitrile (B) using gradient elution conditions: 0–3 min, 65% A/35% B; 3–12 min, 35% A/65% B, 12–13 min, 10% A/90% B; 13–14 min, 0% A/100% B; 14–16 min 0% A/100% B; 16–19 min, 65% A/35% B; and 19–23 min, 65% A/35% B. The following parameters were selected: flow rate, 0.4 mL/min; column incubation temperature, 30 °C; and detection wavelength, 275 nm. The compounds were purified by column chromatography using silica gel Kieselgel 60, 230–400 mesh, 40–63 µm (Merck, Darmstadt, Germany). The purity of the substrate and the yield of the products were assessed by GC using Agilent Technologies 8860 (GC System, Santa Clara, USA) with a flame-ionization detector and carrier gas H_2_ using HP-5 column 30 m × 0.32 mm × 0.25 µm (Agilent, Santa Clara, CA, USA) with the following temperature program: 80 °C (1 min), 320° (30 °C/min) (1 min).

The structures of the compounds were evaluated by NMR techniques. ^1^H NMR, ^13^C NMR, COSY, HSQC, and HMBC spectra were recorded in CDCl_3_ on a Bruker Avance 500 (500 MHz, Billerica, MA, USA) spectrometer. 

HR-ESI–MS/MS analyses of the compounds were performed with RSLC UltiMate 3000 (Dionex, Sunnyvale, CA, USA) coupled with the ESI-Q-TOF, maXis impact mass spectrometer (Bruker Daltonics, MA, USA), and the operating parameters were: flow rate of the sample of 180 µL/min, nebulizer pressure 0.4 bar, the heating gas flow of 3.0 L/min, heating gas temperature of 180/200 °C, data acquisition range of m/z 50–1300/1400 m/z, ionization mode: positive and negative, and ion source energy of 5 eV. 

### 4.2. Microorganisms

The strains used in this study were obtained from the Department of Food Chemistry and Biocatalysis collection at the Wrocław University of Environmental and Life Sciences and from the Institute of Agricultural and Food Biotechnology Collection of Industrial Microbial Cultures (full list available in the Supplementary Materials). The strains were stored on Sabouraud or Czapek agar slants at 4 °C.

#### Molecular Species Identification

We performed DNA analyses for strains with high substrate conversion to confirm proper species identification (based on morphology). Strain KKP3292 was not included in this step, as it was obtained from commercial culture collections and was already identified. DNA from samples AM32, AM91, and AM235 was isolated with a Bead-Beat Micro AX Gravity isolation kit from A&A Biotechnology (Poland, Gdańsk) according to the manufacturer’s instructions. Next, isolated cellular DNA was assessed for its quality (with agarose gel electrophoresis) and quantity (with Qubit 4 fluorometer). An internal transcribed spacer (ITS) fragment was used as a genetic marker in fungal species identification. PCR conditions and sequencing procedures were similar to those described by Hernik et al. [53]. ITS sequences from all analyzed samples were input into the online PROTAX-fungi tool [54] to identify the species.

As we could not determine the identification of the species level for strain AM32 with ITS, we sequenced the whole genome of the strain to aid in additional identification in the future. We used the Oxford Nanopore MinION platform with SQK-LSK110 chemistry and R10.3.1 flow cells and obtained approximately 2 Gb of data for the sample. After sequencing, we used Guppy 6.4.2 (https://community.nanoporetech.com/ (accessed on 4 April 2023) and dna_r10.3_450bps_sup model for base-calling. Basecalled fastq files were then screened for quality with PycoQC [55]. To assemble the genome, we first used Canu v2.2 [56] and then Flye [57,58] and polished assembly with Medaka (https://github.com/nanoporetech/medaka (accessed on 4 April 2023)) to enhance assembly length. Finally, before submission to the NCBI, the obtained data were screened for contaminants using the Foreign Contamination Screen (FCS) tool (https://github.com/ncbi/fcs (accessed on 4 April 2023)) and submitted to the NCBI genome database.

### 4.3. Chemical Synthesis

For this step, 4.3 g (0.023 M) of 3-*n*-butylidenephthalide (Sigma–Aldrich; Saint Louis, MO, USA) and 110 mL of 0.5 M KOH solution in methanol were added to a two-neck round-bottom flask mounted under a reflux condenser. The reaction mixture turned orange. The hydrolysis reaction was carried out at boiling point for 1.5 h. The mixture was cooled, and 10% hydrochloric acid was added until a yellow color appeared. Then, methanol and water were evaporated; 100 mL of THF, 5 mL of 0.1 M KOH aqueous solution, and 0.95 g (0.025 M) of sodium borohydride (Sigma–Aldrich) were added to the residue. The solution again turned orange. The reaction was stirred at 22 °C for 12 h. An aqueous solution of 10% hydrochloric acid was added until a yellow color appeared, and stirring was continued for another 4 h. The solvents were evaporated, ethyl acetate was added, and the reaction mixture was filtered through a 30 cm layer of silica gel placed in a 3 cm diameter column. After complete elution of the product and evaporation of the solvents, 4.0 g of product was obtained (84% yield) with 92% purity according to GC. Compound **1** was purified on silica gel using a mixture of petroleum ether: acetone: ethyl acetate: isopropanol 150:5:5:15 *v/v* as the eluent. 

### 4.4. Biotransformations

#### 4.4.1. Screening Scale Biotransformations

The screening scale biotransformations were performed in 300 mL Erlenmeyer flasks with sterile Sabouraud medium [23], acidified by HCl to pH 5.6. After inoculation, the cultures were incubated for eight days at 25 °C on a rotary shaker. Next, 20 mg of 3-*n*-butylphthalide (**1**) dissolved in 0.5 mL of acetone was added. The samples were collected after 4, 8, and 12 days, extracted using ethyl acetate, and dried with MgSO_4_. After evaporation, they were dissolved in acetonitrile, filtered through a 0.45 µm PTFE filter, and subjected to HPLC analysis.

#### 4.4.2. Preparative Scale Biotransformations

- Method I

Biotransformations with AM91 and AM235 were carried out in the bioreactor–3 L New Brunswick Scientific BioFlo III (Brunswick, Ramsey, MN, USA). The bioreactor with Sabouraud medium was sterilized at 121 °C for 25 min. After sterilization, the temperature was maintained at 20–22 °C, and agitation was set to 75 rpm. A Broadley James D100 Series Oxyprobe was used to measure the level of dissolved oxygen. The air was passed through a sterile 0.2 μm PTFE filter at a 1 L/min flow rate. The previously pre-cultured inoculum (10% *v/v*) was placed into the bioreactor, resulting in a final volume of 1500 mL; 0.4 g of 3-*n*-butylphthalide (**1**) diluted in 5 mL of acetone was added during the exponential phase of growth. The 8-day biotransformation progress was monitored by HPLC. The reaction mixture was divided into three portions (3 × 500 mL), acidified by 0.1 M HCl, washed with brine, and extracted overnight with ethyl acetate (3 × 500 mL) on a laboratory shaker. After extraction, the mixture was centrifuged and evaporated. The crude product was purified by column chromatography using gradient elution—a mixture of petroleum ether: acetone: ethyl acetate: isopropanol 150:5:5:15 *v/v* was initially used and, after substrate **1** was washed, their proportion was changed to 50:5:5:15 *v/v*.

- Method II

In the second method for the preparative scale biotransformation, we used AM32, AM91, AM235, and KKP3292 as the biocatalysts. The microorganisms were grown in 2 L flasks with 500 mL of a sterile Sabouraud medium containing 10% pre-cultured inoculum. After five days of incubation at 25 °C on a rotary shaker, 130 mg of lactone **1** in 0.5 mL was added to the cultures. 

The biotransformations were extracted by ethyl acetate after 12 days for *Penicillium* sp. AM91, *B. cinerea* AM235, and *Botrytis* sp. KKP3292 and, additionally, after 6 days for *Penicillium dierckxii* AM32. The prolonged extraction of the biotransformation mixtures on the laboratory shaker lasted 96 h. After the extraction, the isolation of the products proceeded as described above. 

### 4.5. MAO-A Activity

A MicroCal PEAQ-ITC200 calorimeter (Malvern, Worcestershire, UK) was used to study the interactions of MAO-A with the compounds. The enzyme and compounds were prepared in 1% DMSO with methanol. The measurements were performed at 36.6 °C. The stirring speed was set to 307 rpm, and a total of 19 injections were performed within 50 min, with the reference power set to 10.00 μcal/s. All sample solutions were thoroughly degassed before use. The calorimetric cell was filled with a 20 μM (350 µL) MAO-A sample, and the syringe (2 µL of injection volume) was loaded with a 1 mM/L solution or/and 5-HT (1 mM/L). The ITC data were processed using MicroCal PEAQ-ITC analysis software with calorimetric routines. The following parameters were determined: the standard interaction Gibbs energy (ΔG), standard interaction enthalpy (ΔH), entropy (ΔS), equilibrium constant (K_D_), and stoichiometry of reaction (N) [59].

### 4.6. ADMET Profiling

The pharmacokinetic parameters involving lipophilicity, solubility, and inhibition of CYP isoenzymes CYP1A2, CYP2C9, CYP2C19, CYP2D6, and CYP3A4 were predicted using the Swiss Institute of Bioinformatics tool at the University of Lausanne (http://www.swissadme.ch/index.php (accessed on 25 March 2023)). Toxicity to rat cells was assessed using the Way2Drug platform (http://www.way2drug.com/gusar/acutoxpredict.html (accessed on 25 March 2023)).

## 5. Conclusions

Biotransformations catalyzed by fungi are a useful method to obtain derivatives of 3-*n*-butylphthalide (**1**). Two strains of *Botrytis* and two isolates of *Penicillium* were the most effective in terms of substrate transformation. Both 3-*n*-butylphthalide (**1**) and its metabolites **2**–**4**, obtained via fungal biotransformation, acted as MAO-A inhibitors. Two metabolites, **3** and **4**, were more active than substrate **1**. The lipophilicity for all products, assessed by in silico tests, was in the optimal range, correlating with good permeability and solubility. Contrary to its precursor **1**, the most active 3-*n*-butyl-11-hydroxyphthalide (**3**) had no inhibitory activity on the CYP1A2 isoform; therefore, the possibility of interference with drugs metabolized by this isoform is lower. However, further evaluation of the toxicity of compound **3** is required, as the in silico prediction of this parameter revealed higher toxicity to rat cells than 3-n-butylphthalide (**1**).

Further research may involve tests regarding the reversibility of the process to determine if the usage of phthalide **1** analogs leads to tyramine accumulation, like most commercially available drugs, or has similar actions with moclobemide, a reversible inhibitor of MAO-A.

## Figures and Tables

**Table 1:** Pseudo-molecular ions and fragment ions in ESI-HR-MS analysis of compounds **1**–**4**.

**Compound**	**[M + H]^+^**	**[M + Na]^+^**	**[M + H]^−^**	**Formula**	**Fragment Ions (ESI+) Exp.**	**Fragment Ions (ESI+) Lit.**	**Fragment Ions (ESI-) Exp.** *	**Fragment Ions (ESI-) Lit.** *
1	191.1065	213.0891	-	C_12_H_15_O_2_ C_12_H_14_NaO_2_	145.1012 173.0961	173.0962, 145.1011, 135.0440, 117.0701, 131.0493, 105.0703, 91.0547 [24]; 173.098, 145.103, 135.047, 131.052, 117.072, 105.074, 103.057, 91.057 [21]	-	-
2	-	229.0844	205.0863	C_12_H_14_NaO_3_ C_12_H_13_O_3_	171.0804	189.0908, 171.0805, 153.0700, 145.1011, 143.0855, 133.0285, 117.0700 [24]; 189.091, 171.079, 153.071, 145.102, 143.086, 133.031, 128.064, 117.072 [21]	117.0710 161.0972	161.092
3	-	229.0844	-	C_12_H_14_NaO_3_	171.0804	-	-	-
4	-	-	219.0650	C_12_H_11_O_4_	-	203.0699, 185.0594, 175.0752, 159.0441, 157.0649, 143.0489, 131.0490 [24]; 203.081, 185.070, 175.080, 159.052, 157.075, 143.059, 131.058, 103.060 [21]	131.0866 157.0659 175.0765	175.0755, 157.0652, 131.0853 [24]; 175.078, 157.068, 131.089 [21]

* exp—experimental, lit.—literature source.

**Table 2:** Yields (g per g of the substrate; determined by GC) of compounds **2**–**4** in the biotransformation extracts in relation to the strains used.

Microorganism Strain	Compound 2 [g/g]	Compound 3 [g/g]	Compound 4 [g/g]	Yield of Compounds 2–4 [g/g]
AM32 (6 days)	0.04	0.25	0.53	0.82
AM32 (12 days)	0.01	0.04	0.73	0.77
AM91	0.08	0.27	0.02	0.38
AM235	0.57	0.07	0.02	0.66
KKP3292	0.78	0.02	0	0.80

**Table 3:** Thermodynamic parameters of interactions between MAO-A and compounds **1**–**4**.

Compound	K_D_ (μmol/L)	K_A_ ∗ 10^3^ (L/mol)	∆H (kJ/mol)	∆G (kJ/mol)	∆S (J/mol ∗ K)	IC_50_ (1 μmol/L Inhibitor:1 μmol/L MAO-A)	K*_i_* (μmol/L) K*_M_* 5-HT 0.35 mmol
**1**	24.30 ± 1.39 ^a^	41.15 ± 0.36 ^d^	−4.51 ± 0.02 ^e^	−27.33 ± 1.14	73.85 ± 1.05 ^d^	6.51 ± 2.29 ^l^	241.00 ± 3.55 ^c,d^
**2**	29.90 ± 1.09 ^a^	33.44 ± 0.15 ^a^	−7.77 ± 0.01 ^e^	−26.79 ± 1.55 ^j^	61.55 ± 1.84 ^d^	9.66 ± 0.64 ^l^	994.00 ± 3.18 ^e^
**3**	239.00 ± 4.25 ^c^	4.18 ± 0.35 ^e^	−19.19 ± 0.01 ^f^	−23.49 ± 1.42 ^i^	13.92 ± 0.92 ^a^	1.29 ± 0.59 ^k^	<0.001 ^a^
**4**	67.40 ± 3.15 ^d^	14.83 ± 0.25 ^e^	−13.37 ± 0.02 ^f^	−28.42 ± 1.43 ^j^	48.71 ± 1.05 ^a^	5.45 ± 0.10 ^l^	128.00 ± 1.25 ^c^
**1**, **2**	147.00 ± 3.18 ^b^	6.80 ± 0.15 ^e^	−3.80 ± 0.02 ^e^	−22.69 ± 1.91 ^i^	61.13 ± 1.91 ^d^	45.00 ± 4.44 ^n^	0.08 ± 0.00 ^b^
**1**, **3**	320.00 ± 5.55 ^c^	3.12 ± 0.25 ^e^	−4.63 ± 0.02 ^e^	−20.69 ± 1.52 ^i^	51.97 ± 1.64 ^a^	35.61 ± 3.45 ^n^	200.00 ± 1.18 ^c,d^
**1**, **4**	24.60 ± 1.18 ^a^	40.65 ± 0.33 ^d^	−5.02 ± 0.02 ^e,g^	−27.29 ± 1.33 ^j^	72.07 ± 1.25 ^d^	14.03 ± 1.25 ^m^	716.00 ± 3.95 ^e^
**2**, **4**	20.00 ± 1.45 ^a^	50.00 ± 0.49 ^d^	−5.60 ± 0.01 ^e,g^	−27.84 ± 1.45 ^j^	71.97 ± 1.85 ^d^	5.63 ± 2.45 ^l^	<0.001 ^a^
**2**, **3**	21.70 ± 1.49 ^a^	46.08 ± 0.19 ^d^	−5.27 ± 0.01 ^e,g^	−27.62 ± 1.54 ^j^	72.33 ± 1.08 ^d^	15.02 ± 1.15 ^m^	0.94 ± 0.01 ^b^
**3**, **4**	109.00 ± 2.55 ^b^	9.17 ± 0.52 ^e^	−8.28 ± 0.01 ^e^	−23.74 ± 1.59 ^i^	50.03 ± 1.19 ^a^	2.02 ± 1.19 ^k^	0.92 ± 0.01 ^b^

* Values are expressed as mean value ± SD; n = 3; different letters in one column or no index correspond to significant differences (*p* < 0.05).

**Table 4:** In silico profiling, including lipophilicity (Log P_o/w_), solubility (Log S), and inhibition of the isoenzymes CYP1A2, CYP2C19, CYP2C9, CYP2D6, and CYP3A4 toxicity to rat cells, expressed as LD_50_ [mg/kg], and compliance with the objectives of Lipinski, Ghose, Webber, Egan, Muegge.

Compound	Log P_o/w_	Log S	Inhibition of CYP Isoenzymes	LD_50_ [mg/kg]	Drug-like Properties
**1**	2.81	−2.90	CYP1A2	4872	Lipinski, Ghose, Webber, Egan
**2**	1.92	−2.14	^-^	4582	Lipinski, Ghose, Webber, Egan, Muegge
**3**	1.94	−2.03	^-^	3896	Lipinski, Ghose, Webber, Egan, Muegge
**4**	1.60	−1.99	^-^	2065	Lipinski, Ghose, Webber, Egan, Muegge

## Data Availability

The data presented in this study are available on request from the corresponding author.

## Supplementary Materials

The following supporting information can be downloaded at: https://www.mdpi.com/article/10.3390/ijms241310605/s1.
