# Supplementary material for: Microbial Metabolites of 3-n-butylphthalide as Monoamine Oxidase A Inhibitors
Source: Int J Mol Sci. 2023 Jun 25;24(13):10605. doi: 10.3390/ijms241310605 (PMC10342118; doi:10.3390/ijms241310605)
Supplement: Supplementary file 1 [file ijms-24-10605-s001.zip › ijms-2441838-supplementary.docx]

**Supplementary Materials**

Microbial Metabolites of 3-*n*-butylphthalide as Monoamine Oxidase A Inhibitors

Joanna Gach ^1,^*, Joanna Grzelczyk ^2^, Tomasz Strzała ^3^, Filip Boratyński ^1^ and Teresa Olejniczak ^1,^*

^1^ Department of Food Chemistry and Biocatalysis, Wrocław University of Environmental and Life Sciences, Norwida 25, 50-375 Wrocław, Poland; filip.boratynski@upwr.edu.pl (F.B.)

^2^ Institute of Food Technology and Analysis, Faculty of Biotechnology and Food Sciences, Lodz University of Technology, Stefanowskiego 2/22, 90-924 Łódź, Poland; joanna.grzelczyk@p.lodz.pl

^3^ Department of Genetics, Wrocław University of Environmental and Life Sciences, Kożuchowska 7, 51-631 Wrocław, Poland; tomasz.strzala@upwr.edu.pl

* Correspondence: joanna.gach@upwr.edu.pl (J.G.); teresa.olejniczak@upwr.edu.pl (T.O.)


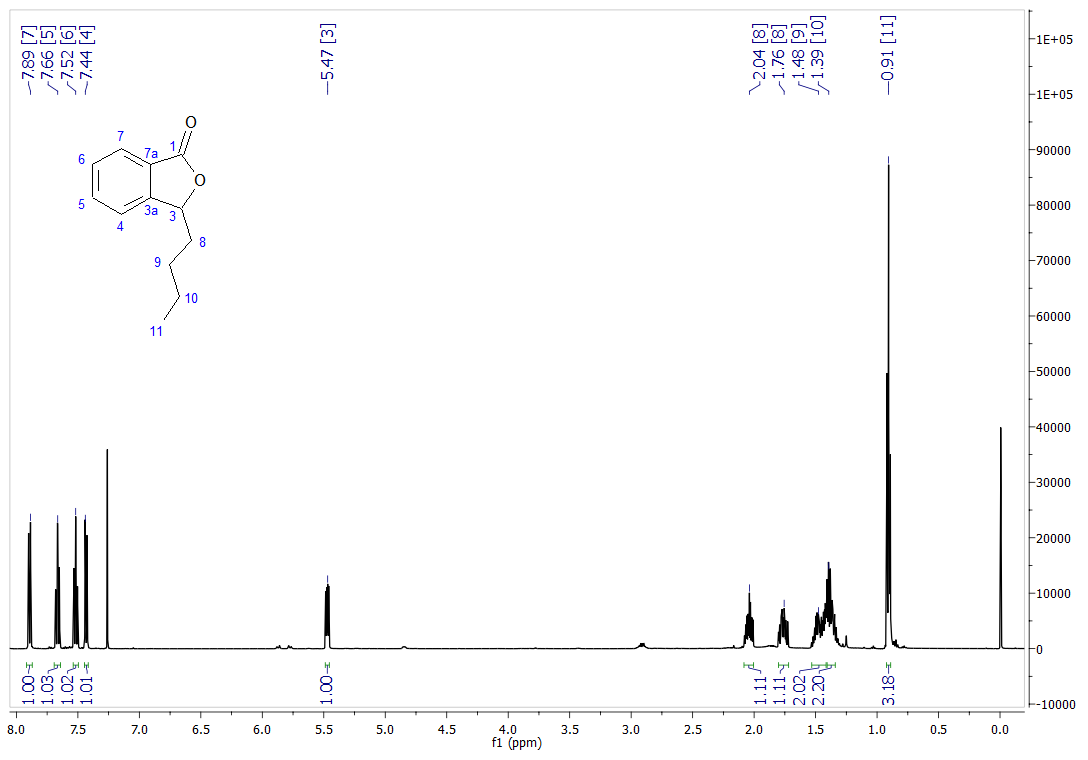


**Figure S1.** ^1^H NMR of 3-*n*-butylphthalide (**1**).


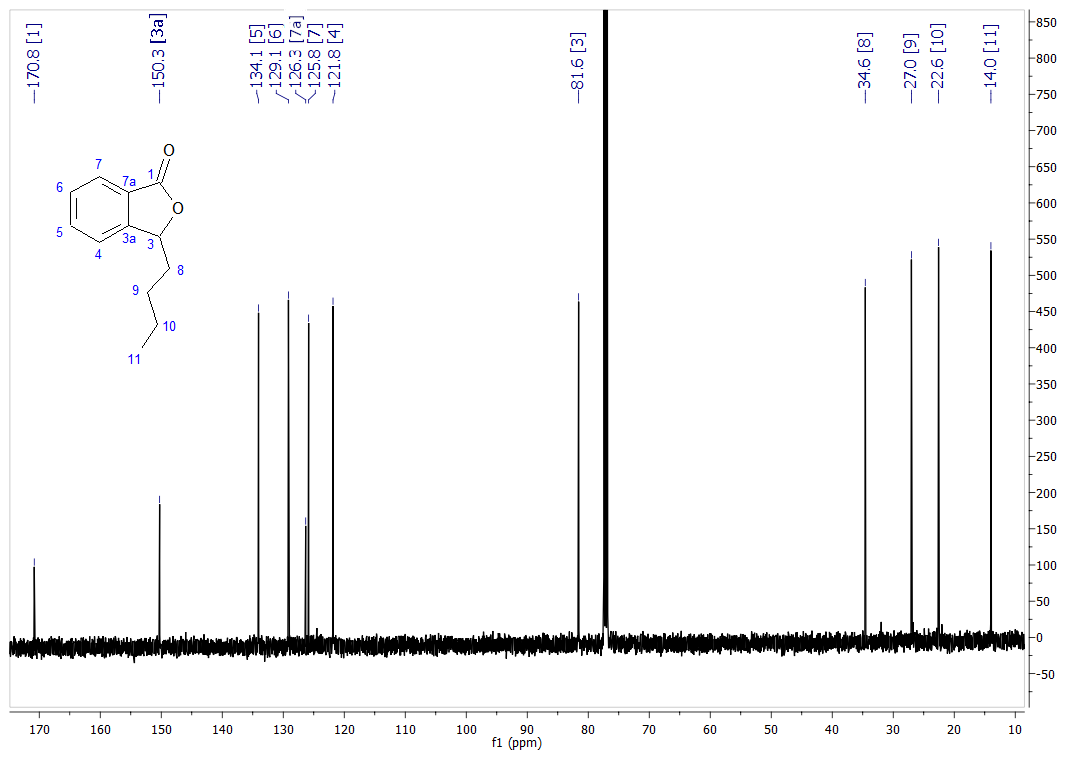


**Figure S2.** ^13^C NMR of 3-*n*-butylphthalide (**1**).

3-*n*-butylphthalide (**1**).

Spectroscopic data: ^1^H NMR (500 MHz, CDCl_3_), δ (ppm): 0.91 (t, 3, *J* = 7.2 Hz, CH_3_-11), 1.31–1.44 (m, 2, CH_2_-10), 1.44–1.54 (m, 2, CH_2_-9), 1.65–1.80 (m, 1, one of CH_2_-8), 1.99–2.12 (m, 1, one of CH_2_-8), 5.47 (dd, 1, *J* = 7.9, 3.7 Hz, CH-3), 7.44 (d, 1, *J* = 7.7 Hz, CH-4), 7.52 (t, 1, *J* = 7.5 Hz, CH-6), 7.66 (t, 1, *J* = 7.5 Hz, CH-5), 7.89 (d, 1, *J* = 7.7 Hz, CH-7).

^13^C NMR (151 MHz), δ (ppm): 14.0 (C-11), 22.6 (C-10), 27.0 (C-9), 34.6 (C-8), 81.6 (CH-3), 121.8 (CH-4), 125.8 (CH-7), 126.3 (C-7a) 129.1 (CH-6), 134.1 (CH-5), 150.3 (C–3a), 170.8 (C-1).

**Figure S3.** ^1^H NMR of 3-*n*-Butyl-10-hydroxy-phthalide **(2)**


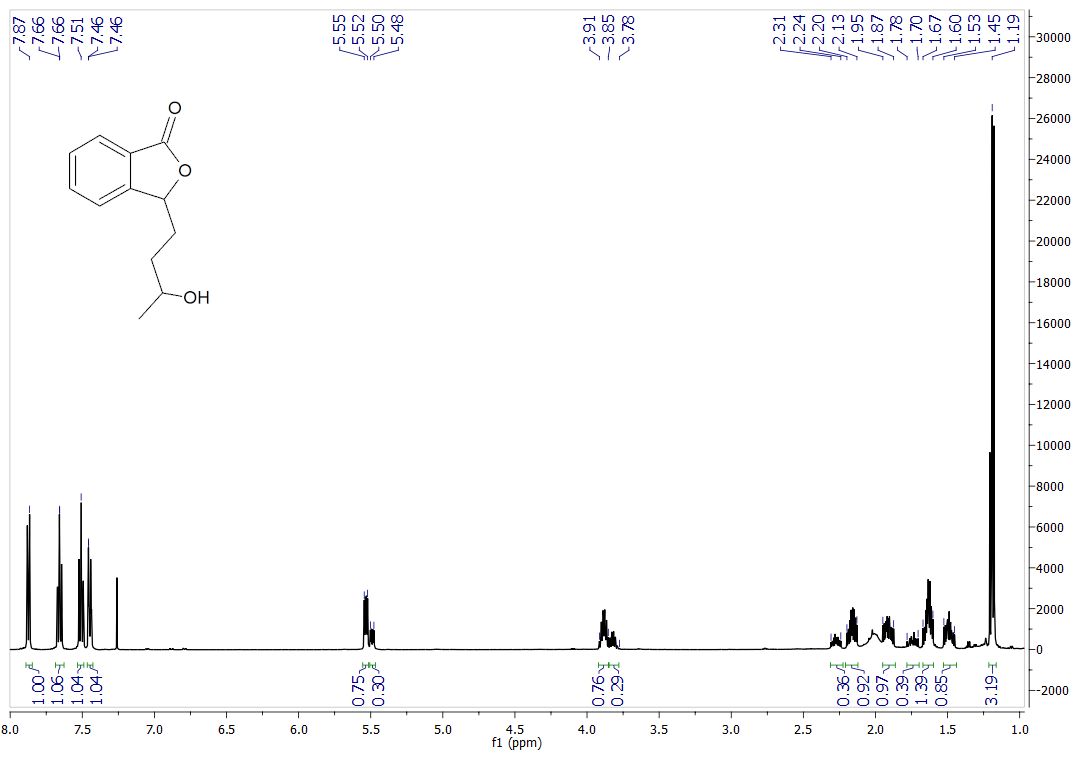

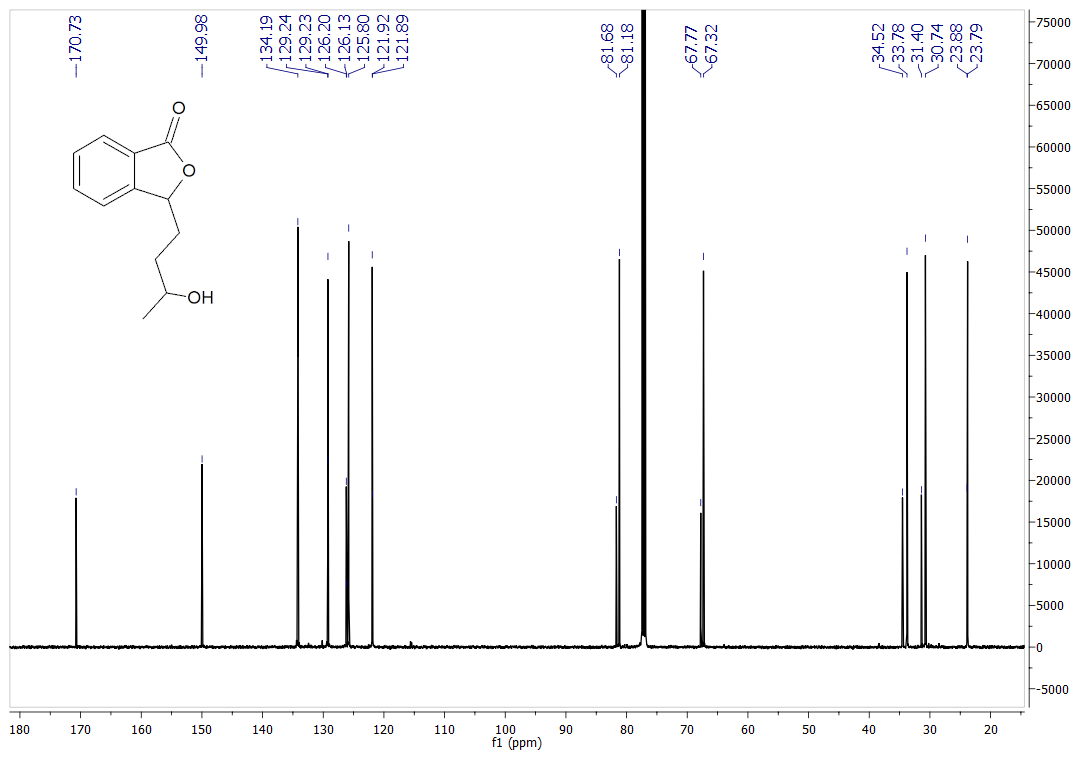


**Figure S4.** ^13^C NMR of 3-*n*-Butyl-10-hydroxy-phthalide **(2)**

**3-*n*-Butyl-10-hydroxyphthalide** (**2**)

Spectroscopic data: ^1^H NMR (500 MHz, CDCl_3_), δ (ppm): 1.19 (dd, 3H, *J* = 8.2, 6.2 Hz, CH_3_-11), 1.44-1.53 (m, 1H, one of CH_2_-9), 1.60-1.67 (m, 1H, one of CH_2_-9), 1.87-1.95 (m, 1H, one of CH_2_-8), 2.13-2.20 (m, 1H, one of CH_2_-8), 3.78-3.85 (m, 1H, CH-10-minor isomer), 3.85-3.91 (m, 1H, CH-10-major isomer), 5.48-5.50 (dd, 1H, *J* = 7.8, 4.0 Hz, CH-3-minor isomer), 5.52-5.55 (dd, 1H, *J* =8.3, 3.8 Hz, CH-3-major isomer), 7.45 (ddd, 1H, *J* = 7.6, 2.8, 0.6 Hz, CH-4), 7.51 (t, 1H, *J* = 7.5 Hz, CH-6), 7.66 (td, 1H, *J* = 7.5, 1.0 Hz, CH-5), 7.87 (d, 1H, *J* = 7.7 Hz, CH-7).

^13^C NMR (151 MHz), δ (ppm): 23.79 (C-11-major isomer), 23.88 (C-11-minor isomer), 30.74 (C-8-major isomer), 31.40 (C-8-minor isomer), 33.78 (C-9-major isomer), 34.58 (C-9-minor isomer), 67.32 (C-10-major isomer), 67.77 (C10-minor isomer), 81.18 (C-3-major isomer), 81.68 (C-3-minor isomer). 121.89 (C-4-minor isomer), 121.92 (C-4-major isomer), 125.80 (C-7), 126.12 (C-7a-minor isomer), 126.20 (C-7a-major isomer), 129.23 (C-6-major isomer), 129.24 (C-6-minor isomer), 134.19 (C-5), 149.98 (C-3a), 170.73 (C-1).


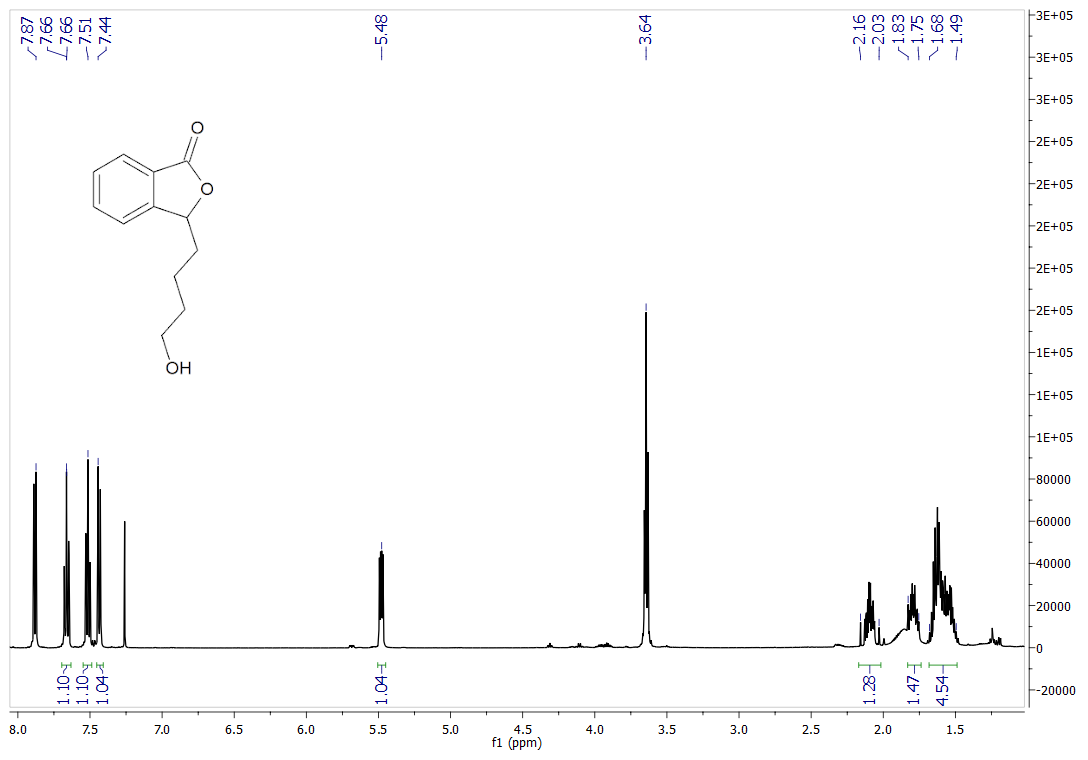


**Figure S5.** ^1^H NMR of 3-*n*-Butyl-11-hydroxy-phthalide **(3)**


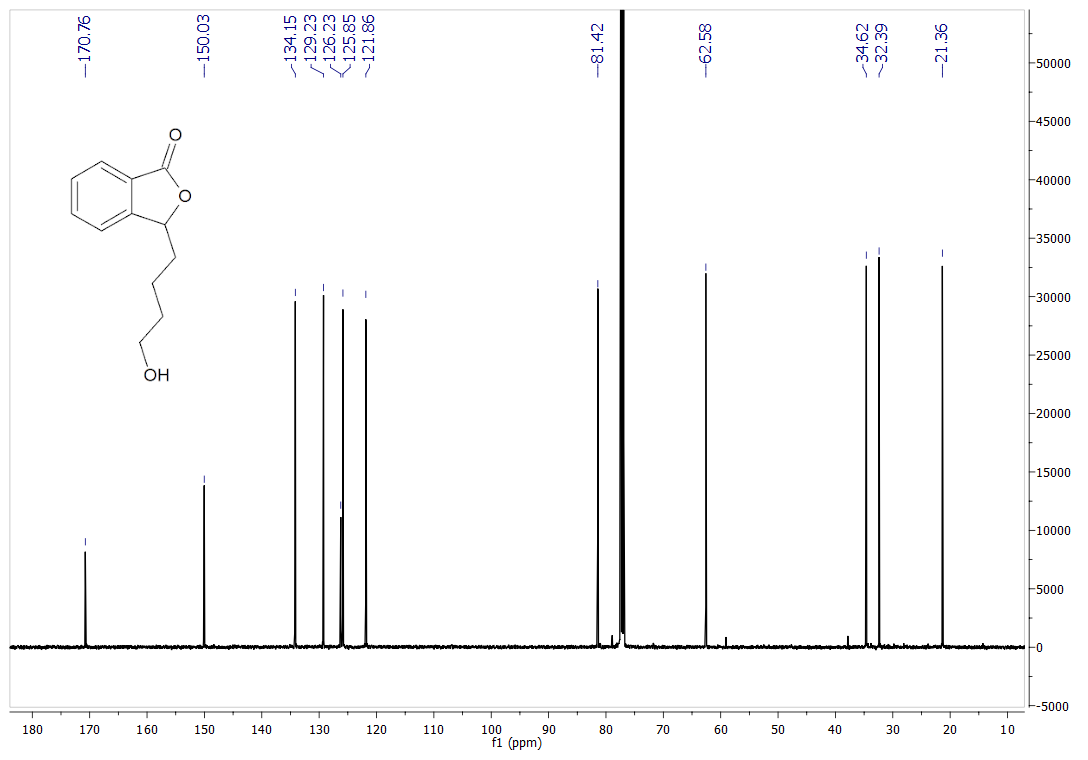


**Figure S6.** ^13^C NMR of 3-*n*-Butyl-11-hydroxy-phthalide **(3)**

**3-*n*-Butyl-11-hydroxyphthalide** (**3**)

Spectroscopic data: ^1^H NMR (500 MHz, CDCl_3_), δ (ppm): 1.49-1.68 (m, 4H, CH_2_-10, CH_2_-9), 1.75-1.83 (m, 1H, one of CH_2_-8), 2.03-2.16 (m, 1H, one of CH_2_-8), 3.64 (t, 2H, *J* = 6.2 Hz, CH_2_), 5.48 (dd, 1H, *J* = 7.9, 4.0 Hz, CH-3), 7.44 (d, 1H, *J* = 7.7 Hz, CH-7), 7.51 (t, 1H *J* = 7.5 Hz, CH-6), 7.66 (td, 1H, *J* = 7.6, 0.8 Hz, CH-5), 7.87 (d, 1H, *J* = 7.6 Hz, CH-7).

^13^C NMR (151 MHz), δ (ppm): 21.36 (C-9), 32.39 (C-10), 34.62 (C-8), 62.58 (C-11), 81.42 (C-3), 121.86 (C-4), 125.85 (C-7), 126.23 (C-7a), 129.23 (C-6), 134.15 (C-5), 150.03 (C-3a), 170.76 (C-1).

**
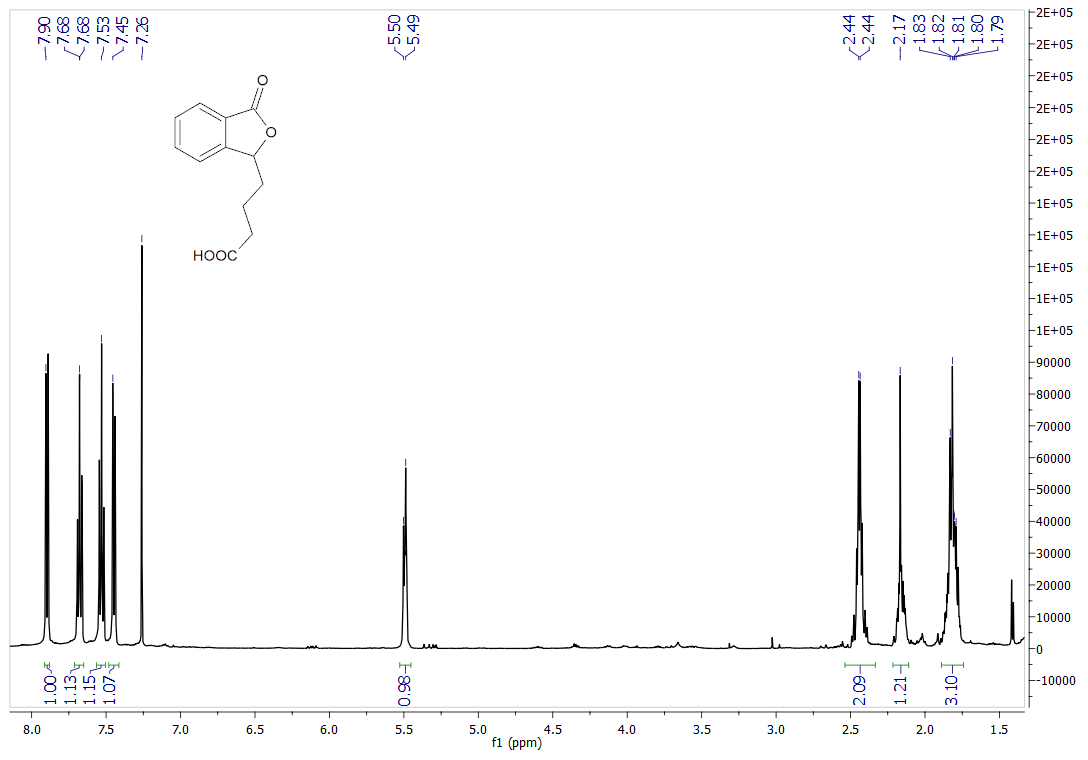
**

**Figure S7.** ^1^H NMR of 3-*n*-butylphthalide-11-oic acid **(4)**


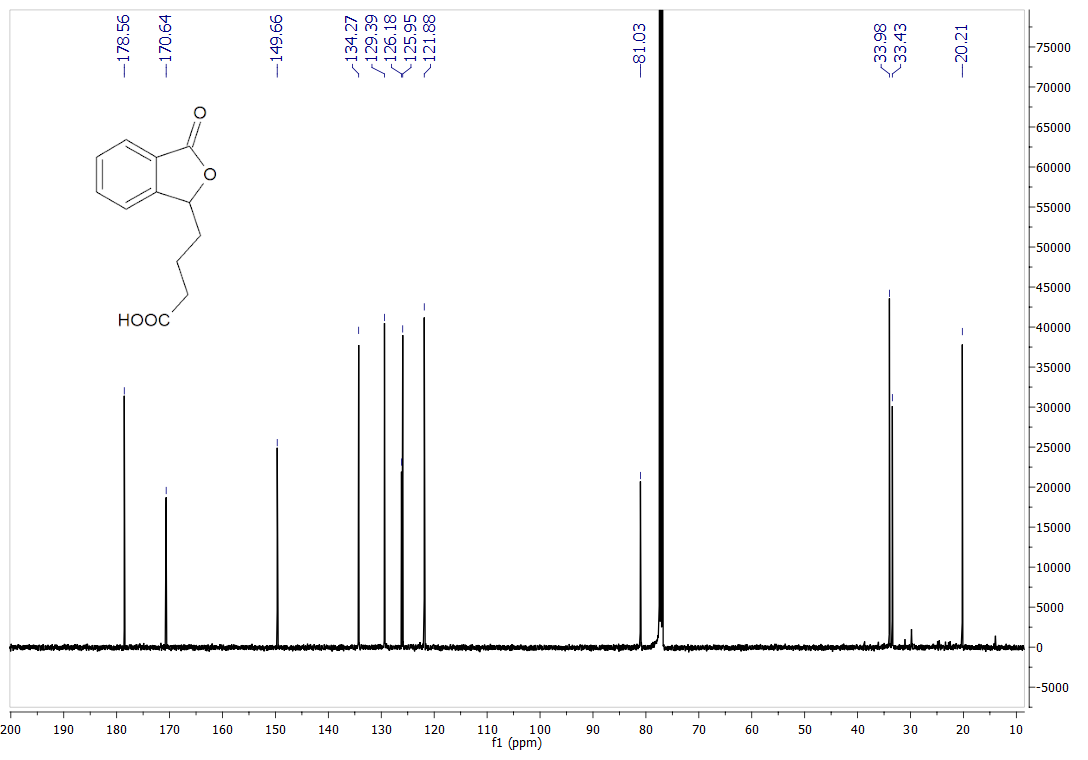


**Figure S8.** ^13^C NMR of 3-*n*-butylphthalide-11-oic acid **(4)**

**3-*n*-butylphthalide-11-oic acid** (**4**)

Spectroscopic data: ^1^H NMR (500 MHz, CDCl_3_), δ (ppm): 1.74-1.88 (m, 3H, CH_2_-9, one of CH_2_-8), 2.10-2.20 (m, 1H, one of CH_2_-8), 2.37-2.50 (m, 2H, CH_2_-10), 5.49 (dd, 1H, *J* = 7.0, 3.5 Hz, CH-3), 7.45 (d, 1H, *J* = 7.6 Hz, CH-4), 7.53 (t, 1H, *J* = 7.5 Hz, CH-6) 7.67 (td, 1H, *J* = 7.6, 0.9 Hz, CH-5), 7.90 (d, 1H, *J* = 7.7 Hz, CH-7).

^13^C NMR (151 MHz), δ (ppm): 20.21 (C-9), 33.43 (C-8), 33.98 (C-10), 81.03 (C-3), 121.88 (C-4), 125.87 (C-7), 126.18 (C-7a), 129.39 (C-6), 134.27 (C-5), 149.66 (C-3a), 170.64 (C-1), 178.56 (C-11).

**
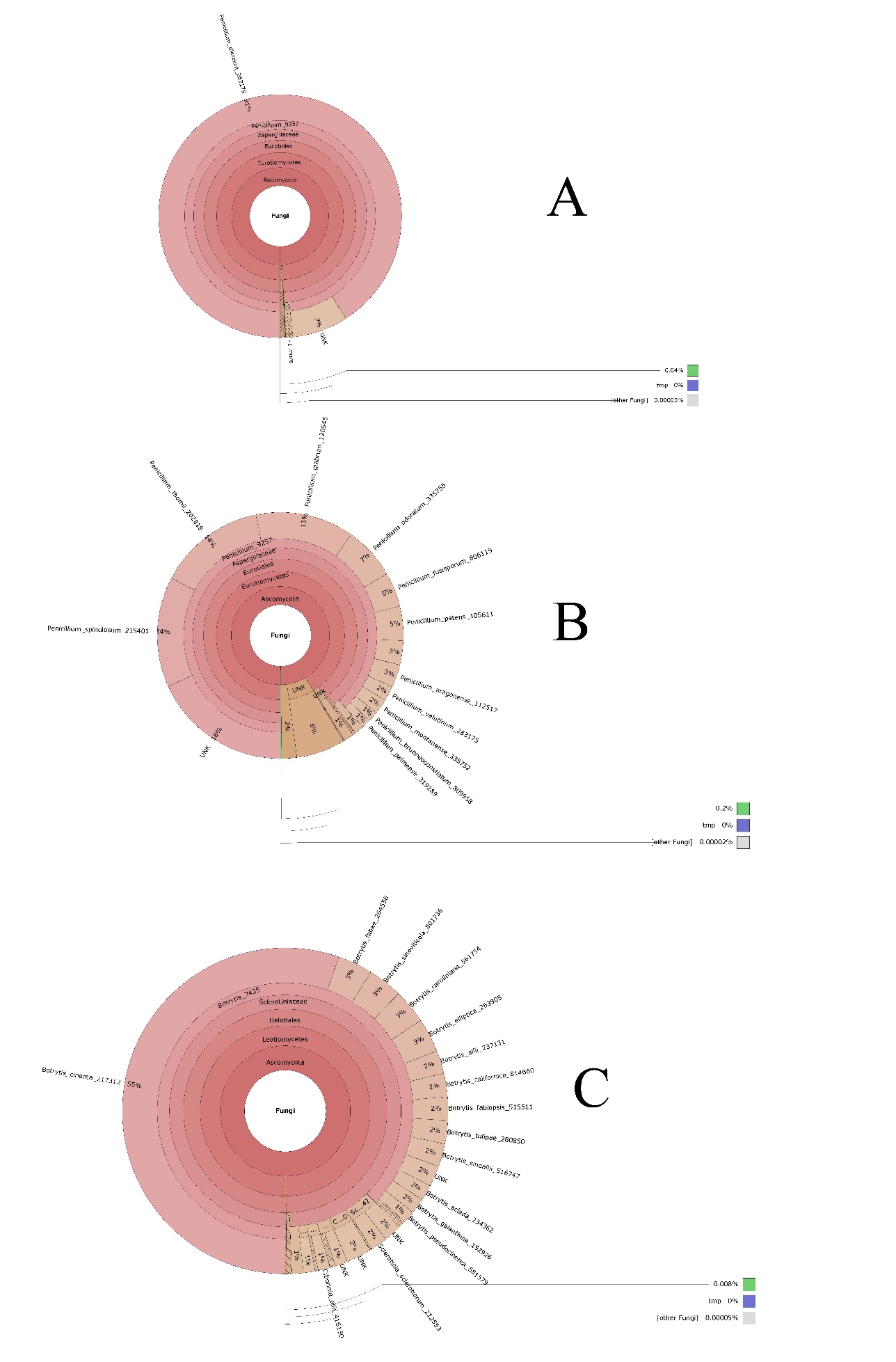
**

**Figure S9.** Species identification results of PROTAX-fungi online tool. A is for strain AM32, B is for strain AM91 and C is for strain AM235.

**Table S1.** Titer (g per liter of culture volume; determined by GC) of compounds 1–4 in the biotransformation extracts in relation to the strains used

| **Microorganism strain** | **Compound 2 [g/L]** | **Compound  3 [g/L]** | **Compound 4 [g/L]** | **Compounds 2-4 [g/L]** |
| --- | --- | --- | --- | --- |
| AM32 (6 days) | 0.01 | 0.06 | 0.14 | 0.24 |
| AM32 (12 days) | 0.00 | 0.01 | 0.19 | 0.20 |
| AM91 | 0.02 | 0.07 | 0.00 | 0.11 |
| AM235 | 0.15 | 0.02 | 0.00 | 0.19 |
| KKP3292 | 0.20 | 0.01 | 0.00 | 0.21 |

**Table S2**. Productivity (g of product per liter of culture volume per hour; determined by GC) of compounds 2–4 in the biotransformation extracts in relation to the strains used

| **Microorganism strain** | **Compound 2 [g/L/h]** | **Compound 3  [g/L/h]** | **Compound 4  [g/L/h]** | **Compound 2-4 [g/L/h]** |
| --- | --- | --- | --- | --- |
| AM32 (6 days) | 0.69 × 10^-4^ | 4.1 × 10^-4^ | 9.7 × 10^-4^ | 16.7 × 10^-4^ |
| AM32 (12 days) | 0.0 | 0.3 × 10^-4^ | 6.6 × 10^-4^ | 6.9 × 10^-4^ |
| AM91 | 0.7 × 10^-4^ | 2.4 × 10^-4^ | 0.0 | 3.8 × 10^-4^ |
| AM235 | 5.2 × 10^-4^ | 0.7 × 10^-4^ | 0.0 | 6.5 × 10^-4^ |
| KKP3292 | 6.9 × 10^-4^ | 0.3 × 10^-4^ | 0.0 | 7.3 × 10^-4^ |

**Full microbial strains list used for the study:**

1. Department of Food Chemistry and Biocatalysis collection at the Wrocław University of Environmental and Life Sciences:

- *Fusarium culmorum* AM 7*,*
- *F. culmorum* AM 9,
- *Papularia rosea* AM 17*,*
- *Acremoniella atra* AM 29,
- Penicillium dierckxii AM 32,
- *Poria placenta* AM 38*,*
- *Sclerophoma pythiopila* AM 55,
- *Piptoporus betulinus* AM 57*,*
- *Penicillium* sp. AM 91*,*
- *Spicaria fusispora* AM 136*,*
- *Chaetomium indicum* AM 158*,*
- *Mortierella isabellina* AM 212*,*
- *Botrytis cinerea* AM 235*,*
- *Fusicoccum amygdali* AM 258*,*
- *Beauveria bassiana* AM 278*,*
- *Armillaria mellea* AM 296*,*
- *Absidia cylindrospora* AM 336,
- *Aphanocladium album* AM 417*,*
- *Verticillium sp.* AM 424*,*
- *Mucor hiemalis* AM 450*,*
- *Armillaria mellea* AM 461*,*
- *Cunninghamella japonica* AM 472*,*
- *Pleurotus ostreatus* AM 482*,*
- *Marasmius scorodonius* AM 497*,*
- *Laetiporus sulphurens* AM 498*,*
- *Pholiota aurivella* AM 522*,*
- *Laetiporus sulphurens* AM 524*,*
- *Trametes versicolor* AM 536*,*
- *Biscognausia marginiata* AM 562

1. Collection of Industrial Microbial Cultures at the Institute of Agricultural and Food Biotechnology:

- *Aspergillus niger* KKP 45,
- *A. niger* KKP 423,
- *A. niger* KKP 424,
- *A. flavus* KKP 686*,*
- *A. flavus* KKP 689*,*
- *Phanerochaete chrysosporium* KKP 784,
- *Trichoderma lignorum* KKP 786*,*
- *Botrytis* sp. KKP 3292
